# Paradoxical effect of baclofen on social behavior in the fragile X syndrome mouse model

**DOI:** 10.1002/brb3.991

**Published:** 2018-04-26

**Authors:** Shimriet Zeidler, Andreea S. Pop, Israa A. Jaafar, Helen de Boer, Ronald A. M. Buijsen, Celine E. F. de Esch, Ingeborg Nieuwenhuizen‐Bakker, Renate K. Hukema, Rob Willemsen

**Affiliations:** ^1^ Department of Clinical Genetics Erasmus University Medical Center Rotterdam The Netherlands

**Keywords:** γ‐aminobutyric acid, baclofen, FMR1, fragile X mental retardation protein, fragile X syndrome, targeted treatment

## Abstract

**Introduction:**

Fragile X syndrome (FXS) is a common monogenetic cause of intellectual disability, autism spectrum features, and a broad range of other psychiatric and medical problems. FXS is caused by the lack of the fragile X mental retardation protein (FMRP), a translational regulator of specific mRNAs at the postsynaptic compartment. The absence of FMRP leads to aberrant synaptic plasticity, which is believed to be caused by an imbalance in excitatory and inhibitory network functioning of the synapse. Evidence from studies in mice demonstrates that GABA, the major inhibitory neurotransmitter in the brain, and its receptors, is involved in the pathogenesis of FXS. Moreover, several FXS phenotypes, including social behavior deficits, could be corrected in *Fmr1* KO mice after acute treatment with GABA_B_ agonists.

**Methods:**

As FXS would probably require a lifelong treatment, we investigated the effect of chronic treatment with the GABA_B_ agonist baclofen on social behavior in *Fmr1* KO mice on two behavioral paradigms for social behavior: the automated tube test and the three‐chamber sociability test.

**Results:**

Unexpectedly, chronic baclofen treatment resulted in worsening of the FXS phenotypes in these behavior tests. Strikingly, baclofen treatment also affected wild‐type animals in both behavioral tests, inducing a phenotype similar to that of untreated *Fmr1* KO mice.

**Conclusion:**

Altogether, the disappointing results of recent clinical trials with the R‐baclofen enantiomer arbaclofen and our current results indicate that baclofen should be reconsidered and further evaluated before its application in targeted treatment for FXS.

## 1 INTRODUCTION

Fragile X syndrome (FXS) is a major X‐linked cause of intellectual disability (ID) and autism spectrum disorders (ASD). Features include developmental delay, deficits in executive functioning, and a wide range of psychiatric problems such as repetitive behavior, social deficits, hyperactivity, anxiety, and irritability (Garber, Visootsak, & Warren, 2008; Hersh & Saul, 2011; Lozano, Rosero, & Hagerman, 2014). In addition, FXS can be accompanied by medical problems, including epilepsy, hyperlaxity, and cardiac disorders (Garber et al., 2008; Kidd et al., 2014). Currently, there is no disease‐modifying therapy available. The most common genetic cause of the disease is a CGG repeat expansion in the 5′untranslated region of the *FMR*1 gene on Xq27.3, leading to hypermethylation of the promoter region and subsequent silencing of the gene (Bell et al., 1991; Oberle et al., 1991; Verkerk et al., 1991). Silencing of transcription of the *FMR1* gene results in the absence of its protein product, the fragile X mental retardation protein (FMRP). FMRP is an RNA‐binding protein that binds approximately 4% of the total brain's mRNA. Many of these mRNAs are coding for proteins important in neuronal connectivity and synaptic plasticity, including PSD95, SAPAP1‐3, α‐CaMKII, Arc/Ar3.1, and Shank1 (Brown et al., 2001; Darnell et al., 2011; Schutt, Falley, Richter, Kreienkamp, & Kindler, 2009). FMRP plays a significant role in transporting these specific mRNAs to the postsynaptic compartment and subsequent local translational control (Willemsen, Levenga, & Oostra, 2011). The *Fmr1* knockout (KO) mouse model displays many phenotypes similar to FXS patients, including ASD‐like behaviors (social behavioral deficits, repetitive behavior), hyperactivity, anxiety, and audiogenic seizures, although many of these phenotypes vary depending on the genetic background of the mouse, the age of the mice, and among research groups (Mineur, Sluyter, de Wit, Oostra, & Crusio, 2002; Moy et al., 2009; Pietropaolo, Guilleminot, Martin, D'Amato, & Crusio, 2011; Spencer et al., 2011).

As many FXS patients manifest social behavior deficits, sleeping disorders, anxiety, and seizures, involvement of the brain's major inhibitory γ‐aminobutyric acid (GABA) pathway has been proposed (D'Hulst & Kooy, 2007). Both the ionotropic GABA_A_ and the metabotropic GABA_B_ receptors have been implicated in FXS and ASD. Studies have demonstrated decreased expression of several GABA_A_ receptor subunits and proteins involved in the metabolism of the GABA neurotransmitter (Adusei, Pacey, Chen, & Hampson, 2010; D'Hulst & Kooy, 2007; Pacey, Heximer, & Hampson, 2009; Paluszkiewicz, Martin, & Huntsman, 2011). The GABA_B_ receptor acts postsynaptically by hyperpolarization of neurons. Presynaptically, GABA_B_ receptor activation leads to reduced glutamate release, resulting in reduced mGluR5 receptor activation and downstream protein synthesis (reviewed in Paluszkiewicz et al., 2011). The combination of hyperactive glutamatergic signaling and the decrease of GABAergic signaling might lead to overall hyperexcitability in FXS, due to an imbalanced excitatory–inhibitory network function (Gibson, Bartley, Hays, & Huber, 2008).

Several studies have now attempted to improve FXS‐related symptoms by correcting this reduced GABAergic activity, using baclofen, R‐baclofen, or arbaclofen. Baclofen is a structural analog of GABA, acting as agonist of the GABA_B_ receptor. It consists of the potent R‐ and the less potent S‐enantiomer, in equal amounts (Sanchez‐Ponce et al., 2012; Silverman et al., 2015). Arbaclofen (STX209) is the more effective R‐enantiomer of baclofen (Lal et al., 2009). Acute arbaclofen treatment corrected several *Fmr1* KO phenotypes, including biochemical, electrophysiological, morphological, and behavioral features (Henderson et al., 2012). Acute R‐baclofen injection showed improvement in social deficits in a version of the three‐chamber sociability test and hyperactivity (Qin et al., 2015). In addition, acute baclofen treatment improved sensory hypersensitivity, electrophysiology, social behavior, and hyperactivity measures (Sinclair et al., 2017). The above‐mentioned results prompted the initiation of a randomized double‐blind placebo‐controlled clinical trial with arbaclofen in 63 children and adults with FXS, resulting in some improvement in post hoc analysis of the most severely affected patients (*n* = 27) (Berry‐Kravis et al., 2012). However, the subsequent larger phase 3 clinical trial with arbaclofen has been terminated prematurely, due to lack of efficacy (Berry‐Kravis et al., 2017).

The assumed involvement of GABA_B_ in social behavior and the fact that chronic treatment might be necessary in the case of FXS prompted us to evaluate chronic baclofen treatment in two social behavioral paradigms: the automated tube test and the three‐chamber sociability test. Unexpectedly, both tests show a paradoxical effect of chronic baclofen treatment on social behavior in *Fmr1* KO mice, worsening their phenotype. Altogether, the latest results of the clinical trial and our current results indicate that baclofen might be questionable as targeted treatment for FXS.

## 2 MATERIALS AND METHODS

### 2.1 Animals

*Fmr1* KO mice (Mientjes et al., 2006) and their WT littermates were bred in house on a C57BL/6 background, using hemizygous or homozygous *Fmr1* KO females and WT males for breeding. Only males were used, because of the X‐linked nature of FXS and because females are known to have a different phenotype in the automated tube test (van den Berg, Lamballais, & Kushner, 2015). Animals used in the automated tube test were housed individually after weaning, 3–4 weeks postpartum, as housing is known to influence the mice behavior (de Esch et al., 2015). For the three‐chamber sociability test, animals were group‐housed with 3–4 males per cage, with a mixed genotype. Stranger mice, which are used in the three‐chamber sociability test, were male age‐matched adult animals of a C57BL/6JRj background (Elevage Janvier, Le Genest‐Saint‐Isle, France). The age of test animals during experiment was between 12 and 16 weeks, when mice reached young adulthood. The mice were weighed, and mice that lost more than 10% of their weight were excluded. Genotyping was performed on toe‐clip at 1 week of age and repeated for validation of the genotype on toe or ear tissue obtained after the behavioral experiments. Animals were kept under standard laboratory conditions with food and water ad libitum and 12‐hr light–dark cycles. Experiments were performed during the light phase at a fixed timeslot. All experiments were approved by the local animal welfare committee. The institutional and national guide for the care and use of laboratory animals was followed.

### 2.2 Behavioral tests

#### 2.2.1 Automated tube test

The automated tube test, adapted from the original tube test (Lindzey, Winston, & Manosevitz, 1961), has been developed to measure hierarchy and social dominance and is often used in ASD research (van den Berg et al., 2015). We used the automated tube test apparatus from Benedictus b.v., a tube made of transparent Plexiglas with a starting box on each side. The protocol has been described earlier (de Esch et al., 2015). In short, the experiment is preceded by 5 days of training and 2 days of rest. The experimenter was blinded for genotype and treatment. Mice were identified using a color code on the tail. Mice were placed in one of the side boxes (starting box) and were motivated to walk to the other side (end box). On the first day, they had to reach the end box within 180 s and the next day within 30 s. On day 2 to 4, the training was repeated for three times on each side. If mice did not leave the starting box within 5 s, a negative stimulus, an air puff, was administered, until the tube was entered. After the training week, the tournament week started. Each day started with two training trials, followed by matches between all mice from both groups. During each match, the designated mice were placed in the starting boxes, one on each side, and the doors were opened. The center door opened when both mice were in its proximity. This was followed by social interaction, and the match terminated when one of the mice walked backward, entering its starting box. The mouse that entered its starting box with all four paws was designated as the loser, while the other mouse was the winner. The group that won most matches was designated as dominant. All matches were repeated on day 2–4 of the tournament week in a different order and a reversed starting side.

#### 2.2.2 Three‐chamber sociability test

The three‐chamber sociability test has been developed to measure social interaction and interest for an unfamiliar mouse compared to an object (empty cage) and is often used in research on neuropsychiatric diseases like ASD (Nadler et al., 2004; Naert, Callaerts‐Vegh, & D'Hooge, 2011). The protocol is a modified version of previous publications that have made use of the three‐chamber setup (Gantois et al., 2013; Nadler et al., 2004). The setup consists of a three‐chamber transparent Plexiglas cage, divided in a center, left and right chambers. The left and right chambers contain a wired cylindrical cage where a mouse previously unknown to the test mouse, the stranger mouse, can be placed. The setup contains two cameras, connected to the ANY‐maze™ Video Tracking System software (Stoelting Co., IL, USA). Prior to the experiment, animals were handled for 2 weeks, to accustom them to the experimenter and the room. On the day of the experiment, animals were acclimated to the room for 30 min. Just before the test, each mouse was habituated to the center chamber for 5 min, followed by 5 min of the sociability test with a stranger mouse placed in the wired cage in the left or right chamber. In the other chamber, an empty cage was placed. The doors were opened, and the mouse could explore all three chambers. The side where the stranger mouse is placed was evenly distributed between genotypes, treatments, and time of day. The movements of the mice were recorded and tracked automatically. Time in direct contact with the stranger mouse or the empty cage, that is, time spent sniffing, was manually scored by the experimenter. Whenever the nose was pointed to the cage and within a predefined circle around the cage, this was considered sniffing. The experimenter was blinded for genotype and treatment throughout the experiment.

### 2.3 Locomotor activity

Locomotor activity was automatically measured using the ANY‐maze™ Video Tracking System software during the three‐chamber sociability test.

### 2.4 Drug treatment

Mice were treated with baclofen (Bufa/Spruyt‐Hillen B.V.) in their drinking water. Aspartame was added to reduce the bitter taste in a concentration of 0.1%. Control water contained aspartame only. Drinking water was refreshed three times a week. It has been previously shown that baclofen crosses the blood–brain barrier (van Bree, Heijligers‐Feijen, de Boer, Danhof, & Breimer, 1991) and that 0.5 mg/ml of arbaclofen, the R‐baclofen enantiomer, reaches acceptable pharmacokinetics and treatment effects in *Fmr1* KO mice (Henderson et al., 2012). Racemic baclofen consists of both the pharmacologically active R‐ and the inactive S‐enantiomer, in equal amounts (Sanchez‐Ponce et al., 2012; Silverman et al., 2015). For that reason, a dose of 1 mg/ml baclofen was used. Based on the increased locomotor activity that was measured in this dose of baclofen, we later reduced the dose to 0.25 mg/ml. *Fmr1* KO mice and WT littermates were treated with baclofen or aspartame drinking water from weaning until the end of the experiment.

### 2.5 Statistical analysis

For the automated tube test, the outcome of each match per mouse was designated as winning or losing. The total number of wins per group was summed up and presented as the percentage of matches won from the total amount of matches of that day. A binomial distribution test was used to determine a significant difference between the total match outcome and the null hypothesis: If both groups are similar, around half of matches are won by each group. To analyze the intrinsic validity of the test, the percentage of matches with a similar outcome to the previous day (i.e., the stability of the test outcome) was calculated (data not shown). A stability >70% compared to the previous day was considered stable.

The three‐chamber sociability test data were analyzed with statistical software (SPSS for Windows, IBM, NY, USA, version 24). Overall effects were evaluated with repeated‐measures analysis of variance (RM ANOVA). Genotype (*Fmr1* KO and WT littermates) and treatment (baclofen with aspartame and aspartame only) were included as between‐subject factor. Stranger side (empty and stranger) was included as within‐subject factor, allowing for correction of time spent sniffing the stranger cage for the time spent sniffing the empty cage. Data are presented as mean ± standard error (SE). For the locomotion analysis, differences between concentrations were calculated with a two‐sided *t*‐test and overall difference was calculated with univariate ANOVA.

## 3 RESULTS

### 3.1 Chronic baclofen treatment (1 mg/ml) worsens the social behavior phenotype in *Fmr1* KO mice and induces aberrant social behavior in WT littermates

#### 3.1.1 Automated tube test

**Figure 1:**
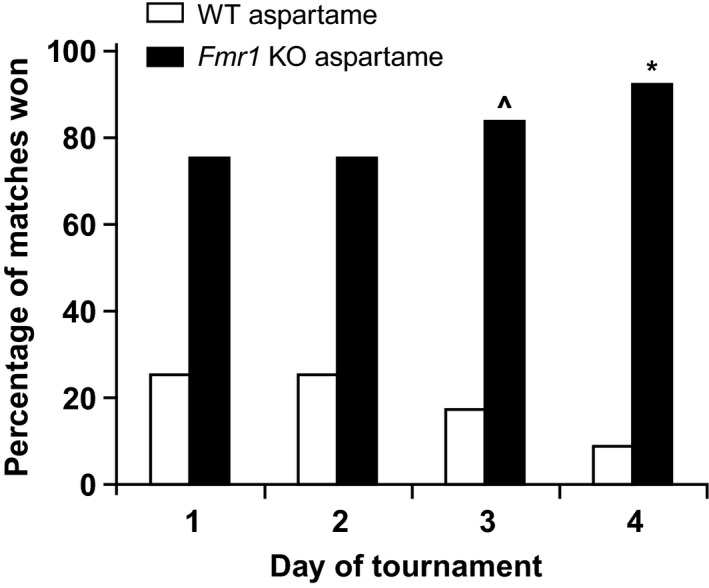
Control experiment of matches between *Fmr1* KO mice and WT littermates using aspartame drinking water, in the automated tube test. *Fmr1* KO mice receiving aspartame drinking water win most of the matches from WT littermates receiving aspartame drinking water (*p* = .054 on day 1 to .003 on day 4, *n* = 4 mice per group). *p* values: ^^^<.05, *<.01, **<.001

As a start, we performed a control experiment to determine whether aspartame in the drinking water affects the outcome of the ATT. These experiments show that *Fmr1* KO mice receiving control aspartame water win most of the matches over their WT littermates receiving control aspartame water (Figure 1). This is indicated as a dominant phenotype and is similar to the phenotype described for *Fmr1* KO mice with normal drinking water (de Esch et al., 2015).

**Figure 2:**
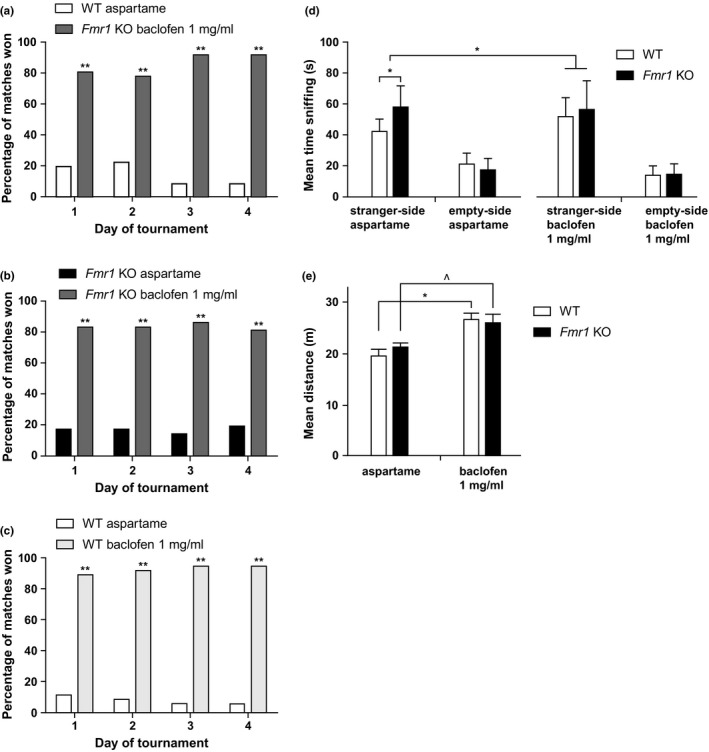
The effect of 1 mg/ml baclofen treatment on the behavior of *Fmr1* KO mice and wild‐type littermates in the automated tube test and the three‐chamber sociability test. (a–c) Results of the automated tube test, expressed in percentage of matches won. (a) *Fmr1* KO mice treated with baclofen win most matches against WT littermates receiving aspartame drinking water, winning 80% of the matches on day 1, which increases to over 90% on day 4 (binomial test, *p* = .000, *n* = 6 per group). (b) *Fmr1* KO mice treated with 1 mg/ml baclofen water win most matches against *Fmr1* KO mice receiving aspartame water, winning more than 80% of the matches (*p* = .000, *n* = 6 per group). (c) WT littermates treated with 1 mg/ml baclofen win most matches against WT littermates receiving aspartame water, winning more than 90% of the matches (*p* = .000, *n* = 6 per group). (d) Sociability behavior, in the three‐chamber sociability test, expressed as average time spent sniffing the stranger mouse, compared to the time spent sniffing the empty cage. All groups sniff the stranger mouse more than the empty cage (two‐way RM ANOVA with within‐subject factor side of stranger, *p* = .000). *Fmr1* KO mice receiving aspartame water sniff the stranger mouse significantly more than WT littermates receiving aspartame water (two‐way RM ANOVA with stranger side as within‐subject factor and genotype as between‐subject factor, *p* < .003). WT littermates receiving baclofen water sniff the stranger mouse more than WT littermates receiving aspartame water (*p* = .008), making baclofen‐treated WT littermates comparable to *Fmr1* KO mice receiving aspartame or baclofen. The average time sniffing the stranger was similar for *Fmr1* KO mice with and without baclofen treatment (RM ANOVA stranger side * treatment, *p* = .869). Data are expressed as mean ± SE; WT aspartame *n* = 13, *Fmr1* KO aspartame *n* = 13, WT baclofen *n* = 11, *Fmr1* KO baclofen *n* = 12. (e) Locomotor activity during sociability phase of the three‐chamber sociability test. WT and *Fmr1* KO receiving baclofen drinking water show increased locomotor activity compared to WT and *Fmr1* KO mice receiving aspartame water (two‐sided independent‐samples *t*‐test, *p* = .001 and *p* = .034, respectively). Data presented as mean ± SE. *p*‐Values: ^^^<.05, *<.01, **<.001

To test whether baclofen treatment affects the dominant *Fmr1* KO automated tube test phenotype, we treated *Fmr1* KO mice with 1 mg/ml baclofen and compared them to WT littermates receiving aspartame drinking water only. Each experiment contained two groups of six mice, resulting in a total of 36 matches per day. We show that *Fmr1* KO mice treated with 1 mg/ml baclofen win most matches from WT littermates receiving aspartame water (Figure 2a), supporting no correction of the phenotype. In the case of a full correction, both groups would win around 50% of matches. In addition, *Fmr1* KO mice treated with baclofen became dominant over *Fmr1* KO mice receiving aspartame drinking water (Figure 2b), in line with worsening of the dominant phenotype. The control experiment of WT littermates receiving aspartame drinking water versus WT littermates treated with baclofen shows that WT mice treated with baclofen (1 mg/ml) become dominant, implying an induced dominant phenotype (Figure 2c).

#### 3.1.2 Three‐chamber sociability test

The time a mouse spent in direct contact with and sniffing the cage where the stranger mouse was placed, or the empty cage, was manually recorded by the experimenter and is depicted in Figure 2d. This measure is expressed as sniffing time and is an indication for sociability (Moy et al., 2009; Nadler et al., 2004). All groups sniffed the stranger mouse significantly more than the empty cage. *Fmr1* KO mice receiving aspartame drinking water show an increased sociability phenotype, that is, sniff the stranger mouse more than WT littermates receiving aspartame drinking water, when corrected for total amount of sniffing. WT mice treated with baclofen showed increased time sniffing of the stranger mouse compared to WT mice receiving aspartame water. This increased sniffing time was similar to average time sniffing of *Fmr1* KO mice receiving aspartame water. The average time sniffing the stranger was similar for *Fmr1* KO mice with and without baclofen treatment.

### 3.2 Chronic baclofen treatment (1 mg/ml) increases locomotor activity

The question addressed in this paper was whether baclofen can reduce abnormal social behavior of *Fmr1* KO mice. Abnormal behavior in mouse models could be confounded with many environmental or intrinsic factors, for example, drug‐induced hyperactivity (Silverman, Babineau, Oliver, Karras, & Crawley, 2013). Because baclofen treatment unexpectedly led to a worsening of the social behavior phenotype in *Fmr1* KO mice in the automated tube test and an induced phenotype in WT littermates in both behavioral tests, we analyzed the locomotor activity which is automatically recorded by the software during the three‐chamber sociability test. The mean distance travelled during the sociability phase is depicted in Figure 2e. Both *Fmr1* KO mice and WT littermates treated with 1 mg/ml baclofen showed a significantly increased locomotor activity compared to littermates receiving aspartame water. The same effect was observed during the acclimatization phase (data not shown).

### 3.3 A lower concentration of baclofen (0.25 mg/ml) shows similar results, without an increase of locomotor activity

Because an increased locomotor activity was observed in mice treated with 1 mg/ml baclofen, we reduced the treatment dose to 0.25 mg/ml baclofen, where no increased locomotor activity was measured and repeated both behavioral tests.

#### 3.3.1 Automated tube test

**Figure 3:**
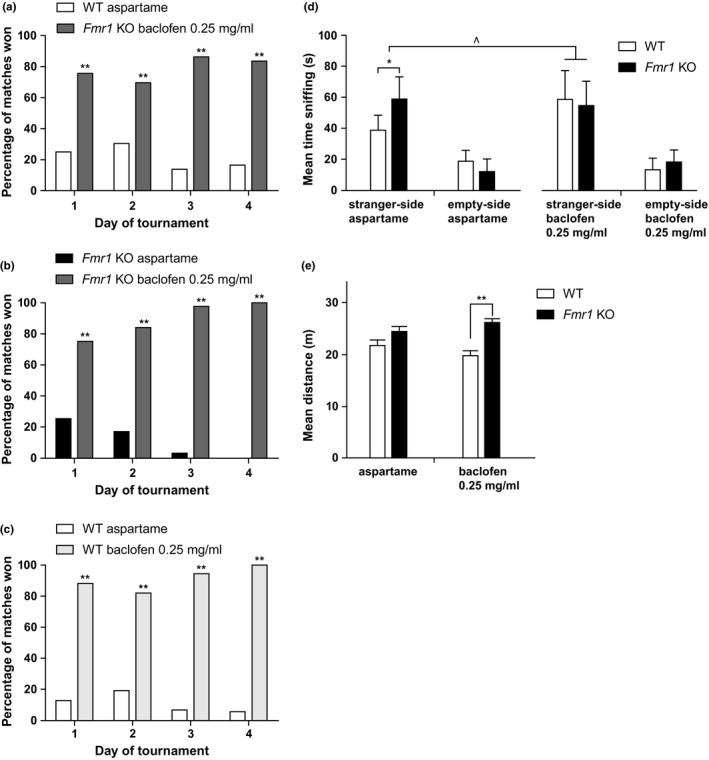
The effect of 0.25 mg/ml baclofen treatment on the behavior of *Fmr1* KO mice and WT littermates in the automated tube test and the three‐chamber sociability test. (a–c) Results of the automated tube test expressed in percentage of matches won. (a) *Fmr1* KO mice treated with 0.25 mg/ml baclofen win most matches against WT littermates receiving aspartame drinking water (binomial test, 70%–90%, *p* = .000, *n* = 6 per group). (b) *Fmr1* KO mice treated with 0.25 mg/ml baclofen water win most matches from *Fmr1* KO mice receiving aspartame water, winning 75% of the matches on day 1, which increases to 100% on day 4 (*p* = .002 on day 1 to *p* = .000 on day 4, *n* = 6 per group). (c) WT littermates treated with 0.25 mg/ml baclofen win most matches from WT littermates receiving aspartame water, winning 80%–100% of matches (*p* = .01 on day 1 to *p* = .000 on day 4, *n* = 4 per group). (d) Three‐chamber sociability test. Sociability behavior, expressed as average time spent sniffing the stranger mouse, compared to the time spent sniffing the empty cage. WT aspartame *n* = 10, *Fmr1* KO aspartame *n* = 10, WT baclofen *n* = 10, *Fmr1* KO baclofen *n* = 10. All groups sniff the stranger mouse more than the empty cage (two‐way RM ANOVA with within‐subject factor side of stranger, *p* = .000). *Fmr1* KO mice receiving aspartame water sniff the stranger mouse significantly more than WT littermates receiving aspartame water (two‐way RM ANOVA,* p* = .004). WT littermates receiving baclofen water sniff the stranger mouse more than WT littermates receiving aspartame water (two‐way RM ANOVA,* p* = .011), making baclofen‐treated WT mice comparable to *Fmr1* KO mice receiving aspartame or *Fmr1* KO mice receiving baclofen. Data are expressed as mean ± SE. (e) Locomotor activity during sociability phase of the three‐chamber sociability test. The locomotor activity does not differ between treatments. *Fmr1* KO mice receiving baclofen have increased locomotor activity compared to WT littermates receiving baclofen (two‐sided independent‐samples *t*‐test, *p* = .000). Data presented as mean ± SE. *p*‐Values: ^^^<.05, *<.01, **<.001

*Fmr1* KO mice and WT littermates were treated with 0.25 mg/ml baclofen or aspartame drinking water. This resulted in a similar effect in the automated tube test, as was seen for 1 mg/ml: *Fmr1* KO mice treated with baclofen win significantly more matches against *Fmr1* KO mice receiving aspartame water (Figure 3b). WT littermates treated with baclofen win most matches against WT littermates receiving aspartame drinking water (Figure 3c). Baclofen treatment did not affect the phenotype in matches between *Fmr1* KO mice receiving baclofen and WT littermates receiving aspartame (Figure 3a). The experiments contained two groups of six mice each, except for the control experiment of WT littermates receiving aspartame drinking water versus WT littermates receiving baclofen drinking water, which contained four mice per group.

#### 3.3.2 Three‐chamber sociability test

Figure 3d depicts the time sniffing for all four groups. All mice sniffed the stranger mouse significantly more than the empty cage. *Fmr1* KO mice receiving aspartame drinking water sniff the stranger mouse more than WT littermates receiving aspartame drinking water. WT mice treated with baclofen showed increased time sniffing the stranger mouse compared to WT mice receiving aspartame water, resulting in an induced phenotype in WT mice. There was no difference in the time spent sniffing the stranger between *Fmr1* KO mice receiving aspartame drinking water or baclofen drinking water. For each group and genotype, 10 animals were used.

#### 3.3.3 Locomotor activity is not increased

The locomotor activity of *Fmr1* KO mice and WT littermates was not increased in mice treated with 0.25 mg/ml baclofen compared with mice treated with aspartame drinking water during the sociability phase (Figure 3e). However, in mice treated with 0.25 mg/ml baclofen, a small but significant difference between *Fmr1* KO mice and WT littermates was measured during the sociability phase, showing higher locomotor activity in *Fmr1* KO mice than WT littermates.

## 4 DISCUSSION

Fragile X syndrome is often characterized by social behavior deficits and autism spectrum features that can significantly impair the patient's social and adaptive abilities. The main goal of our study was to test whether we can correct abnormal social behavior in the FXS mouse model, using baclofen, a GABA_B_ agonist. As FXS is a lifelong disease, and drug treatment is not expected to permanently reverse the features, probably any drug treatment would need to be chronic. For that reason, we investigated chronic baclofen treatment, instead of the often used acute treatment. To measure its efficacy, we used two social behavioral paradigms: the automated tube test and the three‐chamber sociability test. Our results show no improvement in the FXS phenotype after chronic baclofen treatment in both social behavioral tests. As a matter of fact, baclofen seems to increase the dominant phenotype of *Fmr1* KO mice in the automated tube test and even induce aberrant behavior in WT littermates in both behavioral tests. We observed this worsening of the phenotype with two doses of baclofen. The highest dose, 1 mg/ml, was based on pharmacokinetic studies comparing acute intraperitoneal and chronic oral administration of the R‐baclofen‐specific arbaclofen in *Fmr1* KO mice (Henderson et al., 2012). Acute arbaclofen administration corrected several phenotypes. The only data on chronic treatment that was described in this paper regarded a corrected spine phenotype after 12 days of 0.5 mg/ml arbaclofen in drinking water (Henderson et al., 2012). Due to availability issues, we used baclofen, instead of arbaclofen, but corrected for the presence of the less potent S‐baclofen in baclofen, by doubling the dose to 1 mg/ml. We treated our mice for more than 7 weeks. In contrast to their findings for acute treatment, we observed increased locomotor activity after chronic baclofen treatment. This might possibly reflect drug‐induced hyperactivity. This finding led us to hypothesize that the worsening of the social behavior phenotype in the behavioral tests might have been confounded with this drug‐induced hyperactivity. It is known from the literature that drug‐induced hyperactivity can influence social behavior, for example, in the BTBR mouse model of autism (Silverman et al., 2013). For that reason, we repeated the behavioral experiments with a lower dose of 0.25 mg/ml baclofen, where locomotor activity was not increased. However, still a worsening of the social behavioral phenotype and a deleterious effect on WT littermates were observed, independent of the locomotor activity. Of course, we cannot exclude that a dose of 0.25 mg/ml is too small for behavior improvement. Instead, we observe a robust worsening of the behavioral phenotype also for this dose, implying a possible drawback of the use of baclofen.

Hyperactivity is an important hallmark of FXS in patients. Increased locomotor activity in the open‐field test has been described for *Fmr1* KO mice (Ding, Sethna, & Wang, 2014; Mineur et al., 2002; Pietropaolo et al., 2011; Qin et al., 2015; Sørensen et al., 2015; Spencer, Alekseyenko, Serysheva, Yuva‐Paylor, & Paylor, 2005; Yuskaitis et al., 2010). When analyzing the locomotor activity of the three‐chamber sociability test, we did not observe an increased locomotor activity in untreated *Fmr1* KO mice compared with their WT littermates. However, when treated with 0.25 mg/ml baclofen, we observed a small but significant hyperactive phenotype in the *Fmr1* KO mice, compared with their WT littermates. We did not see this phenotype in untreated mice or mice treated with 1 mg/ml baclofen. In the literature, various groups report different findings for locomotor activity in the three‐chamber sociability test. Some papers report no differences between *Fmr1* KO mice and WT littermates (Gantois et al., 2013; Qin et al., 2015), while others show increased locomotor activity in *Fmr1* KO mice (Sørensen et al., 2015). The three‐chamber sociability test is obviously not validated as an open‐field test, and it is not investigated to what extent this locomotor activity is dependent on anxiety, hyperactivity, and other behavioral characteristics. The phenotype we observed in 0.25 mg/ml, but not in 1 mg/ml, could account for increased anxiety or hyperactivity in *Fmr1* KO mice, provoked by baclofen treatment in a dose that does not sedate the animals as a side effect. It is also possible that a higher dose of baclofen influences both WT and *Fmr1* KO mice, resulting in a ceiling effect without further increasing the locomotor activity, while the lower dose slightly affects *Fmr1* KO mice, without affecting WT littermates.

Both baclofen and arbaclofen have been tested in *Fmr1* KO mice and have been shown to reduce FXS‐related phenotypes, such as audiogenic seizures, abnormal spine morphology, synaptic protein synthesis, AMPA receptor internalization, repetitive behavior, and hyperactivity (Henderson et al., 2012; Pacey et al., 2009; Qin et al., 2015; Sinclair et al., 2017). Our results are in a striking contrast with these previously reported papers. This difference could be due to our use of chronic treatment. The only study that used chronic arbaclofen treatment, although administered only for 2 weeks in drinking water, did not assess behavior but only spine morphology (Henderson et al., 2012). This study and other studies have shown a reduction of hyperactivity with acute arbaclofen treatment (Henderson et al., 2012; Sinclair et al., 2017). Another difference with some of the studies is our use of racemic baclofen instead of R‐baclofen or arbaclofen (Sanchez‐Ponce et al., 2012). Unfortunately, it is difficult to assess the optimal dose, as there is no relevant functional biomarker or endophenotype to screen for desirable levels of baclofen in the brain. Furthermore, our results are limited to the social behavioral phenotype, in which the relevance of the GABA_B_ system is unknown.

In addition to FXS, the GABA_B_ pathway has also been implicated in several other neurodevelopmental disorders, including Rett syndrome, Down syndrome, and autism (Deidda, Bozarth, & Cancedda, 2014). The effects of R‐baclofen and arbaclofen have also been studied in mouse models for autism. R‐baclofen has been shown to reverse social deficits and repetitive behavior in two autism mouse models (Silverman et al., 2015). In addition, arbaclofen has been evaluated for patients with ASD. As was the case in the FXS clinical trial, the larger phase 2 randomized placebo‐controlled clinical trial with 150 participants with ASD showed no improvement in the primary outcome measure. However, it did demonstrate improvement of some specific outcome measures in post hoc analyses (Erickson et al., 2014; Veenstra‐VanderWeele et al., 2017). It is currently not known why the promising results in preclinical studies could not be replicated in patients with FXS or ASD. Our results do not support efficacy of chronic baclofen treatment either, possibly revealing drawbacks of baclofen treatment that might be related to the lack of efficacy observed in patients.

In this study, we used two social behavioral tests linked to autism and aberrant social behavior in mice, one of the core features of FXS. Both tests have been shown to be useful in detecting efficacy of targeted treatment, indicating their potential in preclinical screening for therapy. The three‐chamber sociability test is a modified test from Nadler et al. (2004), Naert et al. (2011), developed to measure social interaction and interest for an unfamiliar mouse compared to an object (empty cage), in neuropsychiatric diseases like ASD. Gantois et al. (2013) showed a phenotype similar to ours in the three‐chamber sociability test: increased sociability in *Fmr1* KO mice, compared with WT littermates. The time spent sniffing the stranger mouse is the most important measure for sociability, involving direct and active social approach and interaction (Moy et al., 2009; Nadler et al., 2004). The time in chamber is correlated with the time spent sniffing and could be used as an indirect measurement of social interaction (Gantois et al., 2013; Nadler et al., 2004; Yang, Silverman, & Crawley, 2011). In our experiments, the time spent in the stranger chamber only approached significance, indicating that sniffing time is probably a more reliable measurement of sociability in *Fmr1* KO mice in our setup. Recent publications have also demonstrated enhanced sociability in *Fmr1* KO mice, expressed as time sniffing the stranger mouse (Gantois et al., 2013; Spencer et al., 2005, 2011; Thomas et al., 2011) as well as time spent in the stranger chamber (Gantois et al., 2013; McNaughton et al., 2008; Pietropaolo et al., 2011). However, other studies have demonstrated decreased sociability in *Fmr1* KO mice, which seems more in line with the classic ASD features (Liu & Smith, 2009; Moy et al., 2009; Sinclair et al., 2017). The differences between these studies could be related to differences in genetic background and experimental setup. We modified the three‐chamber sociability test from Gantois et al. (2013), by recording only the first five minutes of the sociability phase, as most of the social approach takes place in this time interval and at that time, the *Fmr1* KO phenotype has been shown to be the most obvious (Gantois et al., 2013; Nadler et al., 2004). Also, we only used the sociability part, as that has been described to be the most sensitive and relevant to autism‐like symptoms (Yang et al., 2011). The second part, preference for social novelty, is more relevant to test social recognition and memory (Yang et al., 2011) and is a less consistent phenotype (Moy et al., 2007, 2009; Pietropaolo et al., 2011).

Our second behavioral test, the automated tube test, is modified from the paradigm developed by Lindzey et al. (1961) to test the effect of different genetic backgrounds on social dominance. *Fmr1* KO mice display a robust dominant phenotype over WT littermates, which can be partially reversed by genetic or pharmacological reduction of the mGluR5 activity (de Esch et al., 2015). In addition, targeting the GABAa receptor using bumetanide has shown a beneficial effect in the tube test as well (Zeidler, de Boer, Hukema, & Willemsen, 2017). Also, for the tube test, conflicting results have been described by different groups (Goebel‐Goody et al., 2012; Spencer et al., 2005), possibly dependent on differences in genetic background, experimental setup, and especially the training protocol and housing conditions (de Esch et al., 2015). In both the automated tube test and the three‐chamber sociability test, the FXS phenotype might be explained by an enhanced sociability, disinhibition in social behavior, increased anxiety, or a combination of those. This fits the observation that FXS patients often do seek social contact and are more empathetic and disinhibited, unlike the social withdrawal often seen in idiopathic ASD (Hall, Lightbody, Hirt, Rezvani, & Reiss, 2010; Steinhausen et al., 2002; Tranfaglia, 2011).

As baclofen is a GABA_B_ agonist, we expected sedation to be a major side effect; hence, we were surprised to find an increased locomotor activity in mice treated with baclofen. Increased locomotor activity could be related to different behaviors in mice such as hyperactivity and anxiety (Kazdoba, Leach, Silverman, & Crawley, 2014) and might be a major confounder on any measurement of social deficits (Silverman et al., 2013). Thus, it is important to address the cause of this paradoxical effect of baclofen. Similar paradoxical effects caused by baclofen have been described earlier in patients with psychiatric diseases or spasticity, as well as in mice (outbred CFW and ICR mouse). Symptoms of elevated mood disorders were observed, such as agitation, insomnia, mania, disinhibition, aggression, or seizures (Dugladze et al., 2013; Geoffroy et al., 2014; Takahashi et al., 2012). Genetic background and pathway characteristics probably play a role in response to baclofen because variations in the GABAergic pathway functioning in different brain regions can ultimately lead to an undesired combined effect. For other GABAergic drugs like benzodiazepines, it has been previously hypothesized that variation in pathway characteristics in the patient's brain leads to differences in the drug response (Bruining et al., 2015; Marrosu, Marrosu, Rachel, & Biggio, 1987; Nardou et al., 2011). Benzodiazepines are known to often cause paradoxical effects in patients with specific neurodevelopmental disorders, especially autism, where the GABAergic pathway has been implicated (Bruining et al., 2015; Mancuso, Tanzi, & Gabay, 2004). In addition, data regarding tolerance induction after chronic administration of baclofen have been reported, although it seems that certain cellular targets of baclofen are more prone to this tolerance induction than others (Lehmann, Mattsson, Edlund, Johansson, & Ekstrand, 2003). The precise mechanism of tolerance induction is not known, as only few studies have focused on chronic systemic baclofen treatment and results in the literature are conflicting. One proposed mechanism is downregulation of the GABA_B_ receptor‐binding sites or phosphorylation of downstream signaling regulators (Keegan et al., 2015; Lehmann et al., 2003; Pacey, Tharmalingam, & Hampson, 2011). In addition, evidence has been published on cross talk between GABA_B_ receptor and mGluR5 receptor after chronic treatment (Pacey et al., 2011). Notably, treating *Fmr1* KO mice acutely with arbaclofen corrects mGluR5‐dependent phenotypes such as AMPA receptor internalization and protein synthesis, underlining the interdependence of both pathways (Henderson et al., 2012). Besides the above‐mentioned variations, also unstable R‐baclofen concentrations due to the short half‐life of the drug are known to contribute to a paradoxical effect (Lal et al., 2009). Because we treated the mice ad libitum with baclofen drinking water, we cannot exclude fluctuations in the blood concentrations. Nevertheless, without quantifying this behavior, mice treated with baclofen in both concentrations did seem to be more agitated or “jumpy.” However, no aggressive behavior was observed. With the lower concentration of baclofen, no increased locomotor activity was measured, but this does not exclude other elevated mood disorder effects on the mice. Probably, aggression, anxiety, mania, or any other behavioral changes, however subtle, could have confounded the social behavior of the mice. We do not know whether our results are due to previously published paradoxical effects of baclofen and whether they are relevant for patients with FXS or other neurodevelopmental disorders. If they are, they predict highly undesirable side effects that may not outweigh any possible improvement. Altogether, this implicates that chronic treatment with baclofen might have a different effect per subject, depending on genetic variation, pathway‐specific differences, and different effects of baclofen per brain region. Moreover, the effect could be dose dependent, dependent on duration of treatment, and be affected by individual differences in metabolism. It would be interesting to unravel these variations and to be able to predict the effect of baclofen in a personalized patient care setting.

In conclusion, treatment of mice with baclofen, a GABA_B_ agonist, resulted in a worsening of the social phenotype in *Fmr1* KO mice and even induction of aberrant social behavior in WT littermates. This deleterious effect might be due to hyperactivity, a paradoxical elevated mood effect, or agitation. Our results on chronic baclofen treatment are in sharp contrast with earlier publications on acute treatment of baclofen or arbaclofen in FXS that have shown benefit on multiple FXS phenotypes. As chronic baclofen treatment worsens the FXS social behavior phenotype of *Fmr1* KO mice and induces aberrant social behavior in WT littermates in these social behavioral tests, the question can be raised whether baclofen is a ubiquitously effective treatment for FXS or might also worsen FXS‐related features in patients. It is unknown whether a similar unexpected effect or paradoxical effect could be relevant in patients with FXS, possibly even in part explaining the disappointing results of the arbaclofen clinical trials. Future research should address whether this effect of chronic baclofen treatment is consistent for other FXS‐related phenotypes and whether it is specific for certain mice strains or patient groups. If baclofen would appear to have a beneficial effect in a subgroup of patients and a deleterious effect in others, the use of baclofen would require identification of predictive factors and a personalized approach.

## CONFLICT OF INTEREST

All authors declare no conflict of interests.
